# Genome-wide analysis of the U-box E3 ligases gene family in potato (*Solanum tuberosum* L.) and overexpress *StPUB25* enhance drought tolerance in transgenic *Arabidopsis*

**DOI:** 10.1186/s12864-023-09890-5

**Published:** 2024-01-02

**Authors:** Zhen Liu, Lei Wang, Yuanming Li, Jinyong Zhu, Zhitao Li, Limin Chen, Hongyang Li, Tianbin Shi, Panfeng Yao, Zhenzhen Bi, Chao Sun, Jiangping Bai, Junlian Zhang, Yuhui Liu

**Affiliations:** 1 State Key Laboratory of Aridland Crop Science, Gansu Agricultural University, Lanzhou, 730070 China; 2 Hebei North University, Zhangjiakou, 075000 China; 3 College of Horticulture, Gansu Agricultural University, Lanzhou, 730070 China; 4 College of Agronomy, Gansu Agricultural University, Lanzhou, 730070 China

**Keywords:** Potato (*Solanum tuberosum* L.), U-box E3 ligases gene family, Expression profiles, Drought stress, Quantitative real-time PCR analysis

## Abstract

**Background:**

Plant U-box (PUB) E3 ubiquitin ligases have vital effects on various biological processes. Therefore, a comprehensive and systematic identification of the members of the U-box gene family in potato will help to understand the evolution and function of U-box E3 ubiquitin ligases in plants.

**Results:**

This work identified altogether 74 PUBs in the potato (StPUBs) and examined their gene structures, chromosomal distributions, and conserved motifs. There were seventy-four *StPUB* genes on ten chromosomes with diverse densities. As revealed by phylogenetic analysis on PUBs within potato, *Arabidopsis*, tomato (*Solanum lycopersicum*), cabbage (*Brassica oleracea*), rice (*Oryza sativa*), and corn (*Zea mays*), were clustered into eight subclasses (C1-C8). According to synteny analysis, there were 40 orthologous *StPUB* genes to *Arabidopsis*, 58 to tomato, 28 to cabbage, 7 to rice, and 8 to corn. In addition, RNA-seq data downloaded from PGSC were utilized to reveal *StPUBs*’ abiotic stress responses and tissue-specific expression in the doubled-monoploid potato (DM). Inaddition, we performed RNA-seq on the ‘Atlantic’ (drought-sensitive cultivar, DS) and the ‘Qingshu NO.9’ (drought-tolerant cultivar, DT) in early flowering, full-blooming, along with flower-falling stages to detect genes that might be involved in response to drought stress. Finally, quantitative real-time PCR (qPCR) was carried out to analyze three candidate genes for their expression levels within 100 mM NaCl- and 10% PEG 6000 (w/v)-treated potato plantlets for a 24-h period. Furthermore, we analyzed the drought tolerance of *StPUB25* transgenic plants and found that overexpression of *StPUB25* significantly increased peroxidase (POD) activity, reduced ROS (reactive oxygen species) and MDA (malondialdehyde) accumulation compared with wild-type (WT) plants, and enhancing drought tolerance of the transgenic plants.

**Conclusion:**

In this study, three candidate genes related to drought tolerance in potato were excavated, and the function of *StPUB25* under drought stress was verified. These results should provide valuable information to understand the potato StPUB gene family and investigate the molecular mechanisms of *StPUBs* regulating potato drought tolerance.

**Supplementary Information:**

The online version contains supplementary material available at 10.1186/s12864-023-09890-5.

## Background

Ubiquitination represents the primary protein modification type within eukaryotes at post-translational level, and it has an essential role in various biological processes [1–4]. The ubiquitination can be mediated by three types of enzymes, receptively, ubiquitin-activating enzyme (E1), ubiquitin-conjugating enzyme (E2), as well as ubiquitin ligase (E3) [5]. First, the ubiquitin molecule forms the thioester bond between a cysteine residue and E1 through the ATP-mediated reaction. Afterwards, activated ubiquitin will be translocated to a conserved cysteine residue in E2 at an active site, and finally E3 will regulate ubiquitin attached to the target protein [6]. Among them, E3 ubiquitin ligase has a major effect on protein ubiquitination, and it identifies target proteins to be modified [7]. Base on the structure, function, and substrate specificity, E3 ubiquitin ligases are divided into 3 main subtypes: U-box, Homologous to E6-associated protein C terminus (HECT), and Really Interesting New Gene (RING) [8]. They mediate the transferring of ubiquitin protein to the substrate by the single-subunit protein or multi-subunit protein [9–11].

The U-box domain, discovered initially within yeast, comprises approximately 75 amino acids [12, 13]. It has a close tertiary architecture to the Ring-finger domain, which lacks classical zinc-chelated histidine and cysteine residues. Therefore, instead of zinc chelation, U-box E3 will use intramolecular interactions for stabilizing the U-box scaffold [14]. The U-box E3 proteins are also contained some classical motifs such as ARM (armadillo repeat region), WD40 repeats, TPR (tetratricopeptide) domain [13, 15], which are involved in protein–protein interactions [16–18].

To date, the U-box E3 proteins (PUBs) have been identified in multiple plant species, with 61 PUBs in *Arabidopsis* [19], 77 PUBs in rice (*Oryza sativa*) [13], 91 PUBs in banana (*Musa acuminata*) [15], 62 PUBs in tomato (*Solanum lycopersicum*) [20], 56 PUBs in grapevine (*Vitis vinifera*) [21], 56 PUBs in pomegranate (*Punica granatum*) [22], 99 PUBs in cabbage (*Brassica oleracea*) [23], 62 PUBs in Chinese white pear (*Pyrus bretschneideri*) [24], 79 PUBs in corn (*Zea mays*), and 125 PUBs in Soybean (*Glycine max*) [25]. As well as, the function of many PUBs has been reported in seed germination [26], root development [27], flowering time [28], hormone signaling [29], self-incompatibility [30], and response to various abiotic/biotic stresses [15, 31, 32].

The PUBs interact with kinase domains and directly control the proteolysis of cellular components, thus, the biological processes in which they participate [29, 33, 34]. Samuel et al. [29] first found that S-locus receptor kinase interacted with and phosphorylated the E3 ligase ARC1 containing U-box/ arm repeats, and ARC1 was a positive regulator of self-incompatibility. Wang et al. [33] found that OsPUB15 can directly interact with the receptor kinase PID2 to regulate plant cell death and resistance to rice blast disease. Antignani et al. [35] found that PUB13 plays an important role in salicylic acid (SA) mediated defense signaling through its interaction with RabA4B and phosphatidylinositol 4-phosphate. In addition, the PUBs can involve in plant responses to abiotic/biotic stresses through various signal transduction pathways, brassinosteroid (BR), gibberellin (GA), and abscisic acid (ABA) [36–38]. Notably, ABA is a major factor regulating drought stress response by inducing stomatal closure to reduce water loss and activating downstream genes in response to drought stress. Previous study found that *PUB12* and *PUB13* regulate the ABI1 (ABA-insensitive 1) level through ubiquitination, in response to ABA [39]. *AtPUB18* and *AtPUB19* negatively regulate ABA-mediated drought stress responses in *Arabidopsis* [32, 36]. Moreover, some PUBs also participate in response to drought stress by ABA-independent pathway. Seo et al. found that AtPUB22 and AtPUB23 are negative regulators of the drought stress response, but their expression not induced by ABA [32]. In Arabidopsis, AtPUB46 and AtPUB48 negatively regulate the tolerance of transgenic plants to drought stress [40]. In summary, PUB proteins are essential in plant response to abiotic/biotic stress.

Potato ranks third place among food crop plants, only second to wheat and rice, and it was first grown in the Andean regions in Bolivia and Peru [41, 42]. Potato cultivation is affected by pathogen invasion and multiple environmental factors, leading to yield loss, and seriously restricting the development of the potato industry. Emerging studies reveal that the Ubiquitination participates in plant responses to biotic and abiotic responses, hormone signaling and various plant developmental pathways [25, 43, 44]. Given the essential role of the U-box gene in plant resistance to abiotic stress, identifying and studying members of the U-box gene family in potatoes will help us understand the evolution and function of the U-box E3 ubiquitin ligase and enrich the gene resources for molecular potato breeding.

## Results

### *StPUB* genes identification and their distributions on chromosomes

We searched the whole potato genome sequence (PGSC_DM_v6.1) for proteins that contain the U-box domain (PF04564) by using HMM (Hidden Markov Model) and BLASTP algorithm. Finally, through adopting SMART conserved domain search tools and NCBI Conserved Domain Data, altogether 74 *StPUBs* were identified (Table S1). The bioinformatics data of StPUB members, such as aa residue number, pI and theoretical MW, were analyzed using the ExPasy site. The StPUB peptides ranged from 140 to 1451 aa, with the molecular weight varied from 15.78 to 162.63 kDa, and the pI value ranged from 5.01 to 9.22. In addition, the StPUB members were predicted to localize in the mitochondria, nucleus, cytoplasm, chloroplasts, or extracellular (Table S1).

**Fig. 1:**
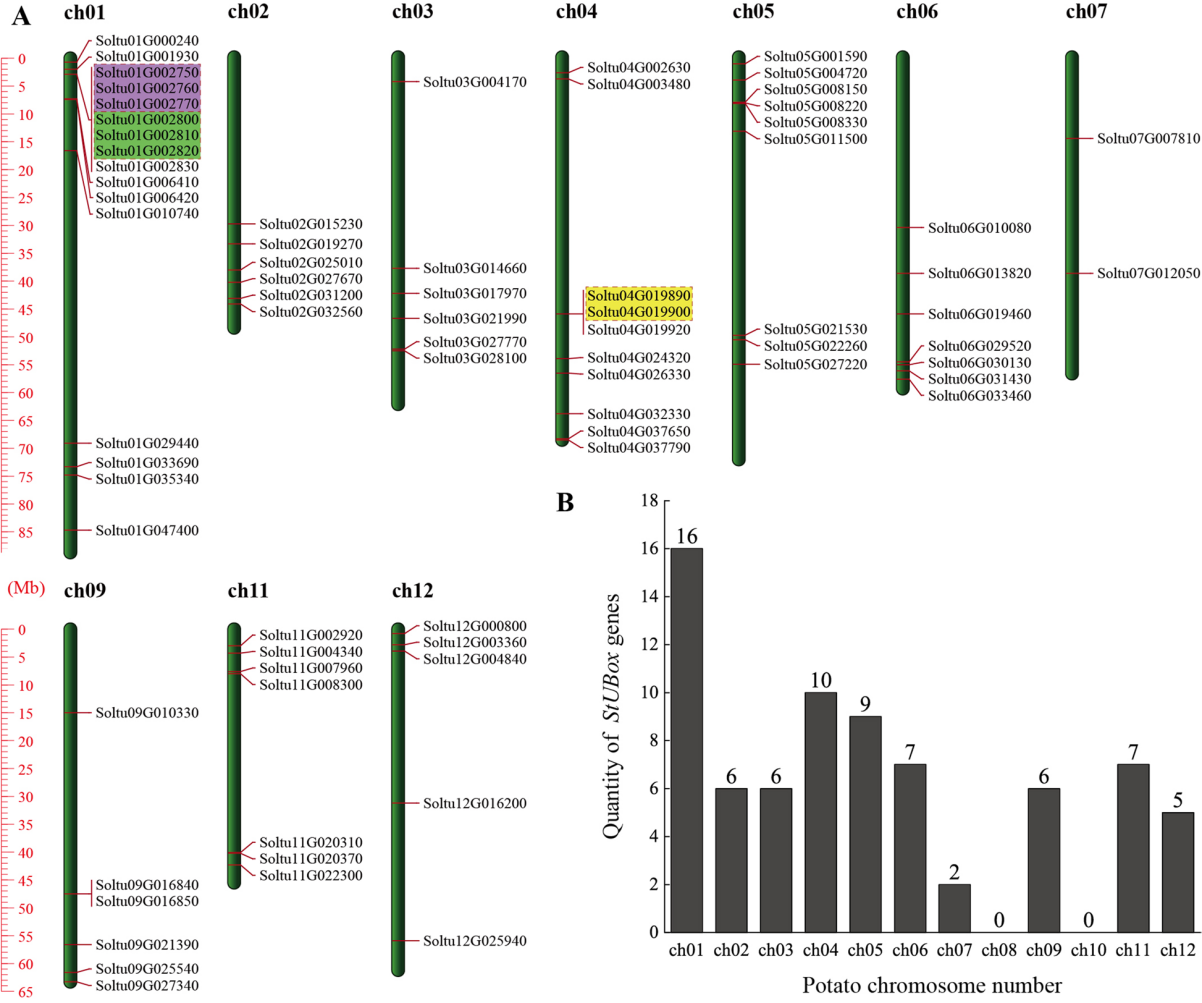
74 *StPUB* gene distributions onto ten chromosomes. **A** “*StPUB* genes distribution map” onto ten chromosomes. Different color boxes denote the tandem duplication genes. **B** *StPUB* gene number on every chromosome

The identified 74 *StPUB* genes showed distribution onto ten chromosomes. (Fig. 1-A). Sixteen *StPUB* genes could be detected on chromosome 1, and it contained most *StPUB* genes. On the contrary, no *StPUB* gene was distributed on chromosomes 8 and 10 (Fig. 1-B). The high densities of *StPUB* genes showed distributions within dismal or proximal ends in the above chromosomes.

### Phylogenetic analysis and classification of StPUBs

**Fig. 2:**
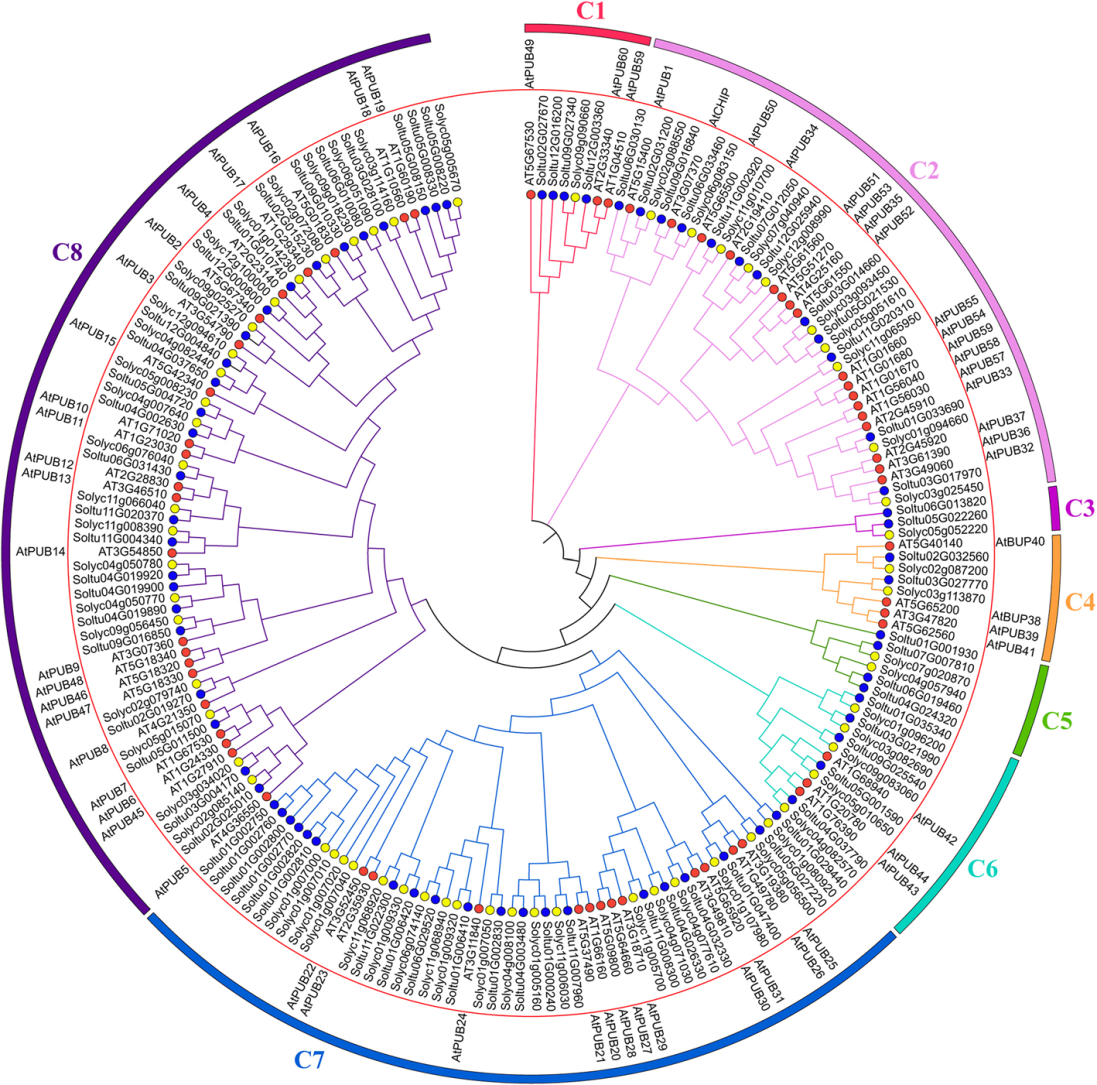
Phylogenetic classification on PUBs in potato, *Arabidopsis*, tomato, rice, cabbage, and corn. Eight subclasses are denoted by diverse colors, separately

For examining evolution relation of PUB genes in potato, tomato, *Arabidopsis*, rice, cabbage, as well as corn, this work used MEGA 7.0 software to build the unrooted phylogenetic tree of 74 potato PUBs (StPUBs), 61 *Arabidopsis* PUBs (AtPUBs), 62 tomato PUBs (SlPUBs), 77 rice PUBs (OsPUBs), 79 corn PUBs (ZmPUBs), and 99 cabbage PUBs (BoPUBs) sequences (Fig. 2). All of 451 PUBs were divided into eight subclasses, namely C1- C8 (Table S1). Among them, the C1 subclass has the least number of PUB members, including one StPUB, one AtPUB, two BoPUBs, three ZmPUBs, and three OsPUBs. The C8 subclass has the most significant number of PUB members, with 26 StPUBs, 22 SlPUBs, 24 AtPUBs, 36 BoPUBs, 25 ZmPUBs, and 22 OsPUBs.

### StPUB gene structures along with conserved motifs

**Fig. 3:**
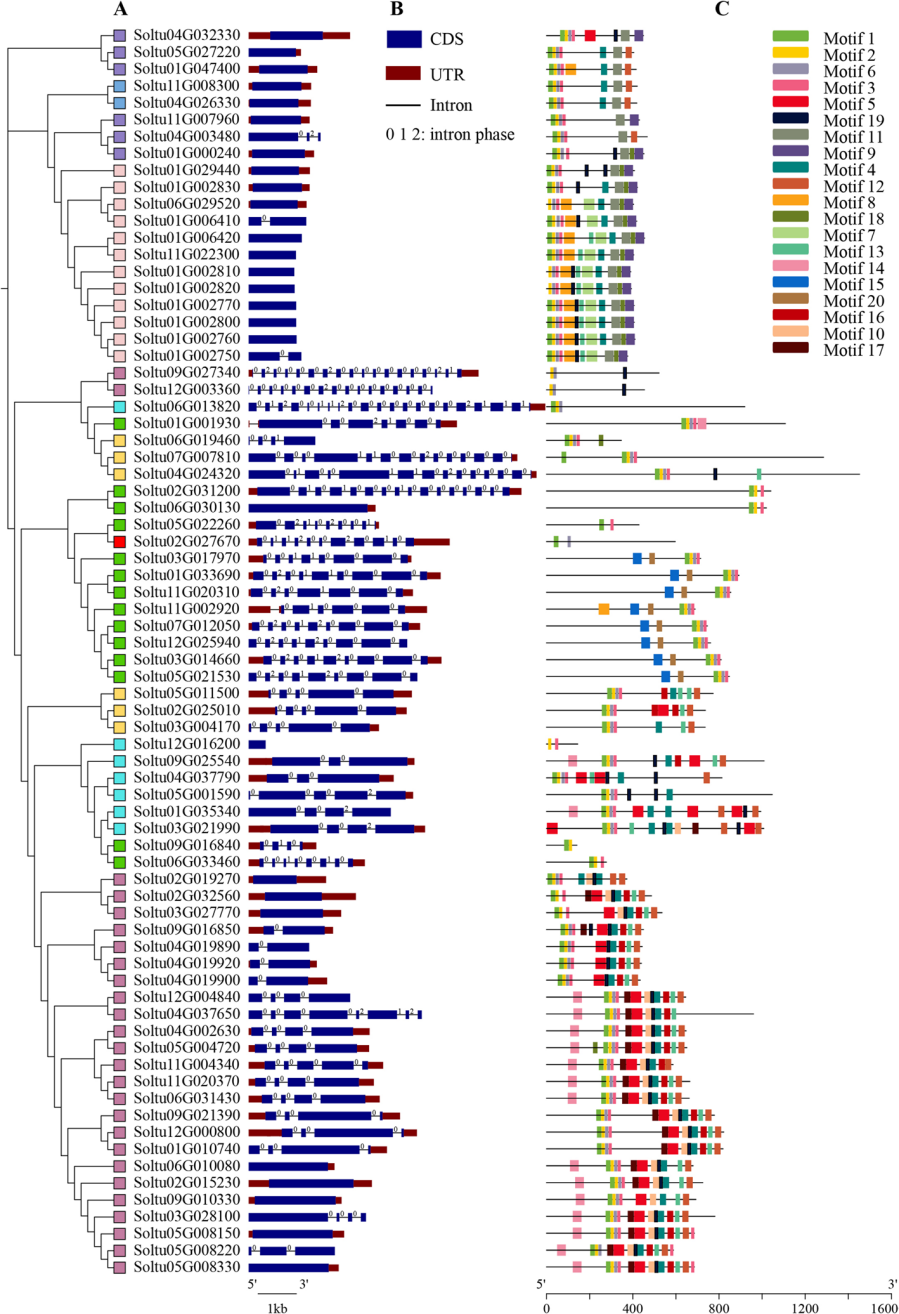
Analysis of StPUBs from the perspectives of phylogenetic relations, gene structures, and conserved motifs. **A** StPUBs phylogenetic tree. Boxes of different colors represent eight subclasses of StPUBs, the red box represents the C1 subclass, the yellow box represents the C2 subclass, the green box represents the C3 subclass, the blue box represents the C4 subclass, the purple box represents the C5 subclass, the light pink box represents the C6 subclass, the light blue box represents the C7 subclass, and the khaki box represents the C8 subclass, respectively. **B** *StPUB* genes’ intron/exon organization. The black line with an identical length and the blue box indicates the intron and exon, respectively, whereas the red box indicates the upstream/downstream area. Specifically, 0, 1, and 2 stand for the intron splicing phase. **C** Conserved motif distributions within StPUBs. MEME program was employed for predicting candidate motifs. Those 20 boxes with diverse colors stand for 20 different putative motifs. Supplementary Table 2 presents detailed information on 20 candidate motifs

By adopting MEGA 7.0 software, this study adopted full-length aa sequences from 74 StPUBs for constructing the unrooted phylogenetic tree. According to phylogenetic analysis on six plant species shown in Fig. 2, 74 StPUBs were also classified into eight subclasses (Fig. 3-A). Overall, the results of the two evolutionary analyses were relatively similar, for example, members of the C7 and C8 subclasses were consistent across the two analyses. But there are some differences, such as the members of the subclasses C1, C2, C3, and C4 were staggered in the phylogenetic analysis of the potato.

To study the structure of *StPUB* genes, the full-length cDNA sequence was compared to the corresponding gDNA sequence to determine the exon–intron structure in every *StPUB* gene. As a result, the exon number within *StPUB* genes was 1 to 21. Among them, 26 (35.14%) genes were intronless, six (8.11%) genes contained one intron, five (6.76%) genes contained two introns, 13 (17.57%) genes contained three introns, five (6.76%) genes contained four introns, and 19 (25.68%) contained more than five introns (Fig. 3-B and Table S1).

To better understand StPUBs diversification, MEME 5.4.1 program was used to identify conserved motifs in 74 StPUB proteins, and 20 predicted motifs were annotated. Table S2 displays detailed information on 20 motifs. As a result, some subfamilies have unique motif distribution, Motif 7, motif 8, motif 9, motif 11, and motif 18 were unique to subclass C7. Motif 17 was presented only in subclass C8. Motif 5 and motif 10 were specifically contained in subclass C4, C6, and C8. Motif 15 and 20 were distributed in subclass C2 (Fig. 3-C and Table S2). The U-box domain comprised motif 1, motif 2, motif 3, and motif 6. Motif 1 and motif 2 were mainly presented in C-terminal within the U-box domain, whereas motif 3 and motif 6 mainly occurred in N-terminal within the U-box domain. In addition, 31 out of those 74 StPUBs (41.89%) contained ARM domains, all belonging to C2, C7 and C8 subclasses. 26 StPUBs (35.14%) only contained the U-box domain, whereas 17 StPUBs (22.97%) contained other domains, such as WD40, TRP, STYKc, and Ufd2P.

To analyze whether these motifs were conserved in other plants, the special conserved motif in potato, tomato, *Arabidopsis*, cabbage, rice, and corn were identified using the MEME website (Table S3 and Fig. S1). We found that motif 1, motif 2, motif 3, motif 4, motif 6, motif 8, motif 9, and motif 10 identified in StPUBs were relatively conserved in potato, tomato, *Arabidopsis*, cabbage, rice, and corn. The motif 1, motif 2, motif 3, and motif 6 make up the U-box domain, and motif 4, motif 5, and motif 10 make up the ARM domain. The results indicated that the U-box domain and ARM domain were conserved during evolution in different species.

**Fig. 4:**
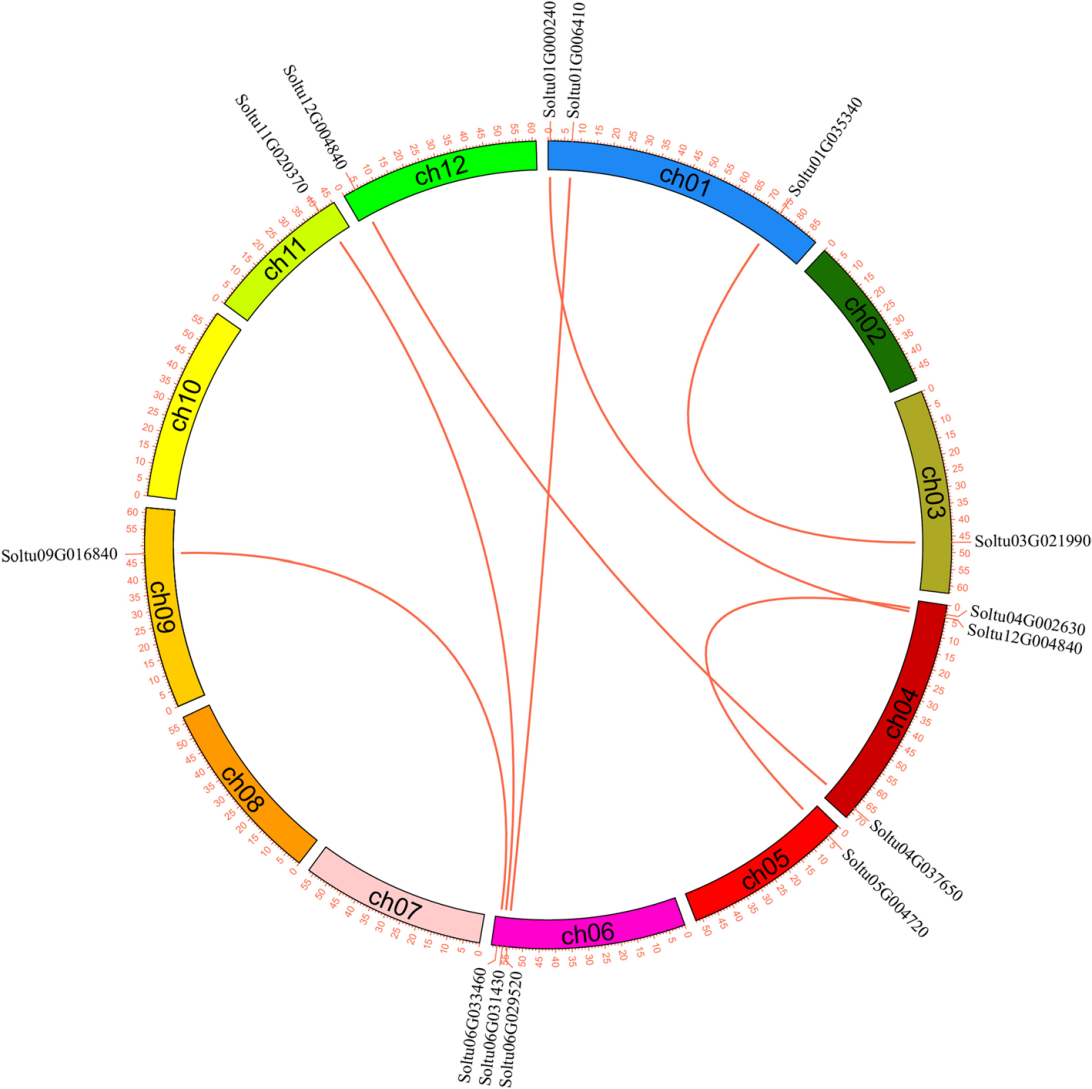
Segmental replication events for *StPUB* genes in potato. The red lines indicate segmental duplication of *StPUB* genes

Gene duplication is a primary driving force for plant evolution. To reveal the StPUB gene family expansion mechanism, gene duplications were analyzed using the MCScanX program. We identified 5 pairs (8 *StPUB* genes, 10.81%) of tandem duplications genes, of which chromosomes 1 had four pairs of tandem duplication genes (*Soltu01G002750*/*Soltu01G002760*, *Soltu01G002760*/*Soltu01G002770*, *Soltu01G002800*/*Soltu01G002810*, and *Soltu01G002810*/*Soltu01G002820*), and chromosomes 4 has one pair of tandem duplication genes (*Soltu04G019890*/*Soltu04G019900*) (Fig. 1-A). Seven pairs of genes (14 *StPUB* genes, 18.92%) were segmentally duplicated (Fig. 4 and Table S4). As a result, some *StPUB* genes might be produced through gene duplication events. The tandem and segmental duplication had critical effects on StPUB gene family expansion.

**Fig. 5:**
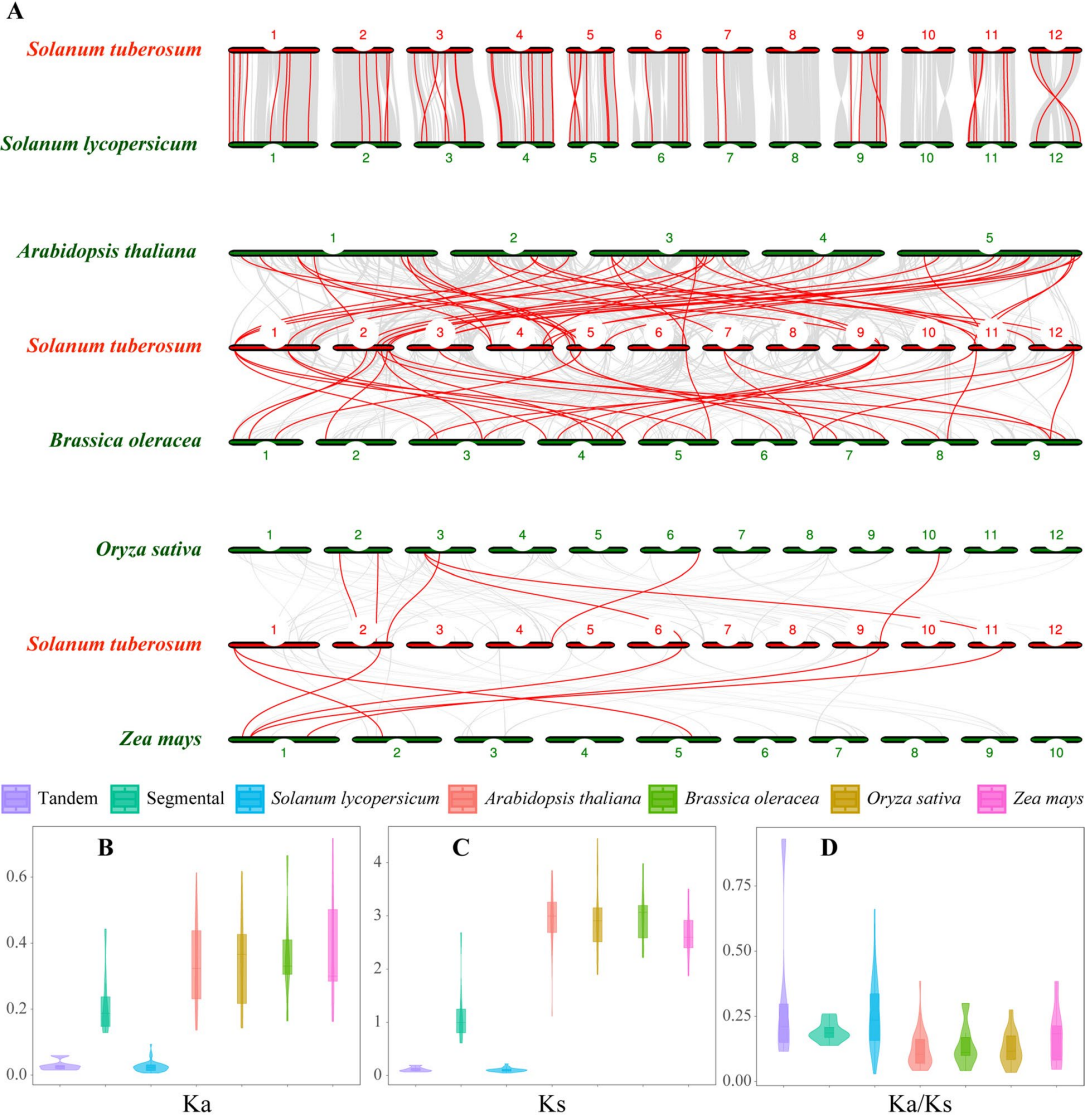
Orthologous relations of StPUB genes against tomato, *Arabidopsis*, cabbage, rice, and corn. The red line represents the orthologous relationship of the StPUB gene to tomato, *Arabidopsis*, cabbage, rice, and corn (A). Average values of Ka, Ks, and Ka/Ks (B-D), respectively, of duplicated genes. The horizontal axes stand for tandem duplication (Tandem), segmental duplication (Segmental) in potato, and the orthologous genes between potato and another plant

Ks and Ka substitution rates are the foundation for assessing duplication events’ positive selection pressure. The Ka and Ks for each duplicated *StPUB* gene pair were determined by KaKs Calculator 2.0 program. As a result, tandem duplication had Ka/Ks of 0.1155–0.9295, and that of segmental duplication was 0.1378–0.2590 (Fig. 5 and Table S4). For all duplication events, Ka/Ks was < 1, indicating that the *StPUB* gene family might have evolved under purifying selective pressure.

For studying StPUB gene family evolution, synteny relations between PUB genes in potato, tomato, *Arabidopsis*, cabbage, rice, and corn were analyzed using the MCScanX program. This work discovered altogether 58 ortholog pairs in potato versus tomato, the Ka/Ks ranged from 0.0291 to 0.6608 (Fig. 5 and Table S5). There were 40 pairs of orthologs were identified between potato and *Arabidopsis*, with Ka/Ks of 0.0415–0.3842 (Fig. 5 and Table S6). A total of 28 pairs of orthologs between potato and cabbage, the Ka/Ks ranged from 0.0346–0.2747 (Fig. 5 and Table S7). There were seven pairs of orthologs in potato and rice, with Ka/Ks of 0.0412- 0.3002 (Fig. 5 and Table S8). Another eight pairs of orthologs between potato and corn were identified, and the Ka/Ks ranged from 0.0464 to 0.3837 (Fig. 5 and Table S9).

### *StPUB* gene expression patterns within various tissues in DM potato

**Fig. 6:**
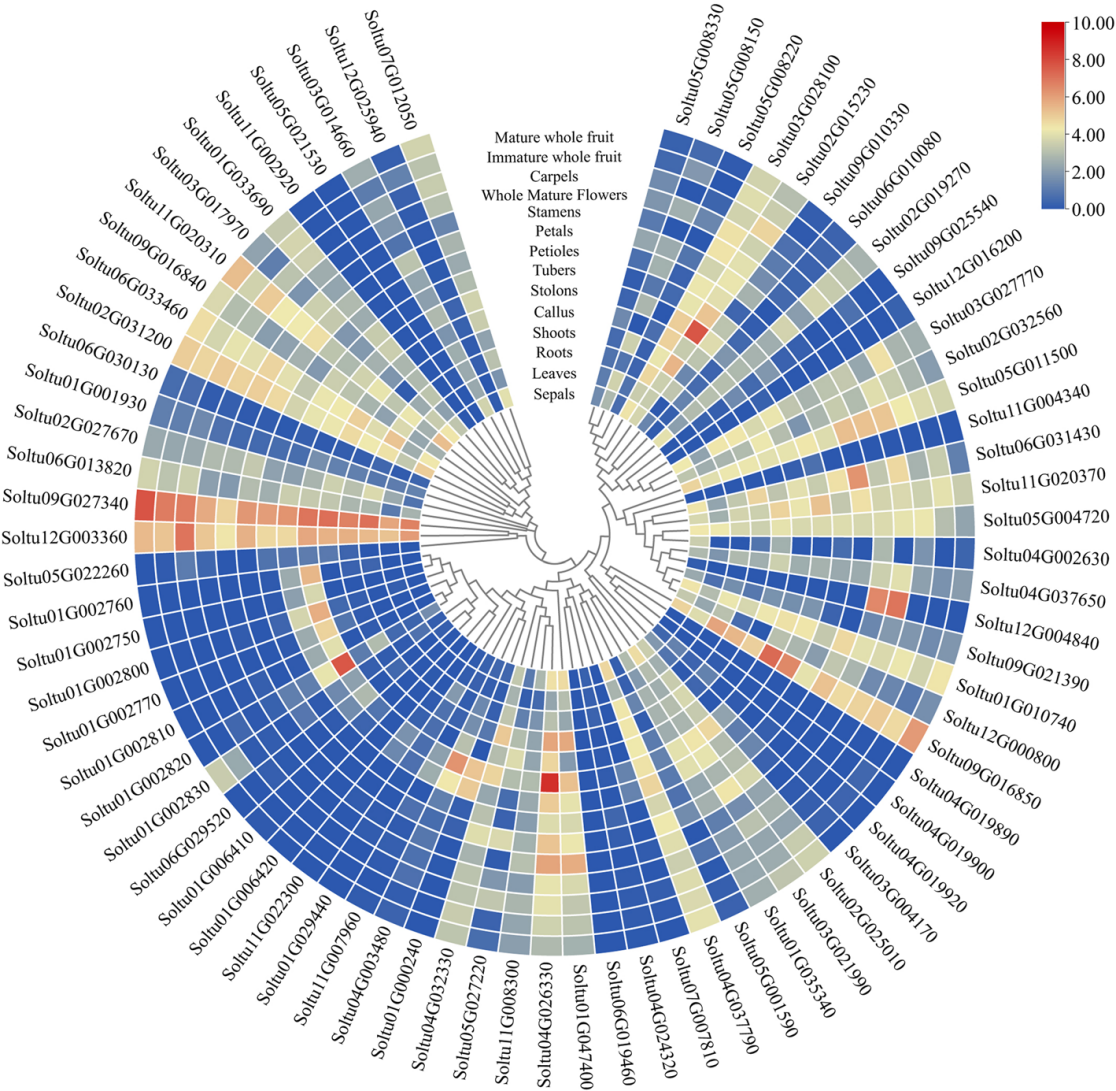
StPUB gene expression patterns within diverse tissues (sepals, leaves, shoots, roots, tubers, stolons, stamens, petioles, callus, carpels, petals, mature flowers, immature fruit, mature fruit) were analyzed according to transcriptome data. In the heatmap, colors represent FPKM values determined based on the binary logarithm

For understanding *StPUB* genes expression profiles in specific tissues, this work examined StPUB gene expression levels within 14 DM potato tissues (sepals, leaves, shoots, roots, callus, stolons, tubers, petioles, petals, stamens, carpels, whole mature flowers, immature whole fruit, mature whole fruit) using the transcriptome data downloaded from PGSC (Fig. 6 and Table S10). The results showed that 11 *StPUB* genes (*Soltu09G016850*, *Soltu11G020370*, *Soltu04G037790*, *Soltu02G031200*, *Soltu05G011500*, *Soltu02G015230*, *Soltu12G003360*, *Soltu11G020310*, *Soltu09G027340*, *Soltu03G028100*, and *Soltu01G010740*) were highly expressed (FPKM > 5) within every tissue type. Whereas three *StPUB* genes (*Soltu04G019890*, *Soltu11G002920*, and *Soltu06G019460*) were not expressed (FPKM = 0). Some *StPUB* genes were denoted in specific tissues, for instance, *Soltu01G002800* showed special up-regulation within stolons and tubers (FPKM > 12). *Soltu12G004840* was particularly expressed in stamens and whole mature flowers (FPKM > 94). Nine StPUB genes (*Soltu01G002800*, *Soltu02G015230*, *Soltu01G000240*, *Soltu04G003480*, *Soltu01G002750*, *Soltu04G026330*, *Soltu01G002760*, *Soltu01G002770*, and *Soltu01G002820*) were specifically expressed in stolons, of which three *StPUB* genes (*Soltu01G002800, Soltu01G000240,* and *Soltu04G003480*) were also highly expressed in tubers. Notably, eight genes only contained U-box domains, except Soltu02G015230, which contained both the U-box and ARM domain.

### *StPUB* genes expression analysis under abiotic stresses and hormone treatments in DM potato

**Fig. 7:**
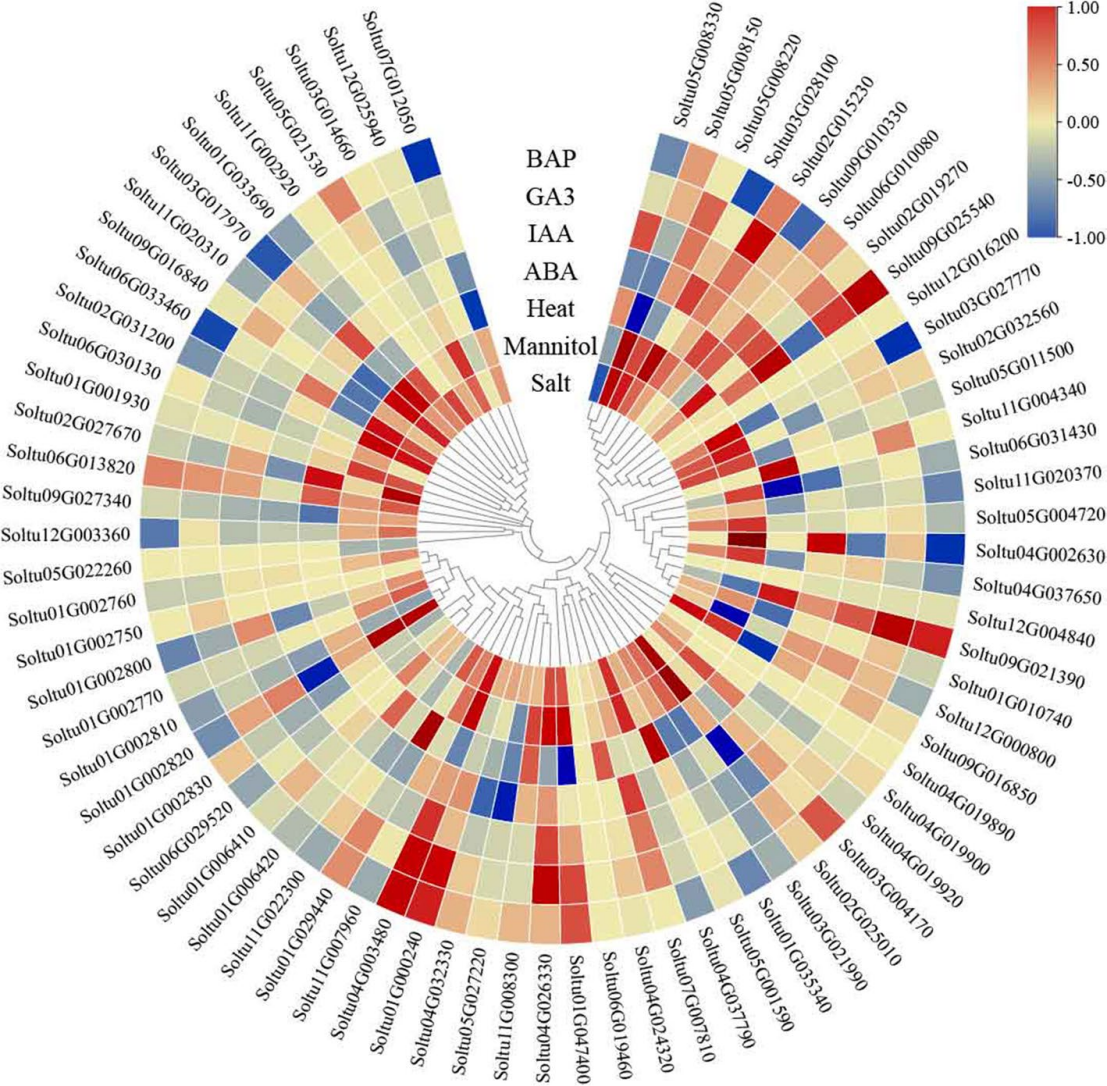
*StPUB* gene expression patterns within DM potato treated by abiotic stresses (salt, mannitol, heat stress), and hormones (BAP, ABA, IAA, GA3). The color represents the value using log_2_FC

For investigating abiotic stress responses in *StPUB* genes, *StPUB* gene expression profiles treated with salt (150 mM NaCl, 24 h), drought (Mannitol 260 uM, 24 h), and heat (35℃, 24 h) stresses were analyzed using the PGSC-derived RNA-seq data. As a result, there were 11, 24, and 12 *StPUB* DEGs (FPKM > 1 and |log_2_FC|> 1) under the above three stresses, separately. Two *StPUBs* (*Soltu09G016850* and *Soltu05G008150*) were differentially expressed (FPKM > 1 and |log_2_FC|> 1) under the above three abiotic stresses. Ten *StPUBs* were differentially expressed under two abiotic stresses. A total of 21 *StPUB* genes only responded to one single stress (FPKM > 1 and |log2FC|> 1) (Fig. 7 and Table S10).

For better exploring alterations of *StPUB* gene levels with diverse hormone treatments (ABA: 50uM, 24 h; IAA: 10uM, 24 h; GA3: 50 uM, 24 h; BAP: 10 uM, 24 h), the expression patterns of 74 *StPUB* genes were investigated with the use of the RNA-seq data. As a result, 6, 3, 6, and 11 *StPUB* DEGs (FPKM > 1 and |log_2_FC|> 1) were detected under ABA, IAA, GA3, and BAP treatments, separately. Among them, three *StPUBs* (*Soltu03G017970*, *Soltu04G002630*, and *Soltu09G025540*) were up-regulated under ABA treatment, two *StPUBs* (*Soltu05G008330* and *Soltu04G003480*) were up-regulated under IAA treatment, six *StPUBs* (*Soltu02G015230*, *Soltu01G029440*, *Soltu01G000240*, *Soltu09G021390*, *Soltu04G003480*, and *Soltu04G026330*) were up-regulated under GA3 treatment, and 4 *StPUBs* (*Soltu01G029440*, *Soltu01G000240*, *Soltu09G021390*, and *Soltu04G003480*) were up-regulated under BAP treatment (Fig. 7 and Table S10).

### Expression of three *StPUB* genes in potato plantlets under NaCl and PEG treatments

**Fig. 8:**
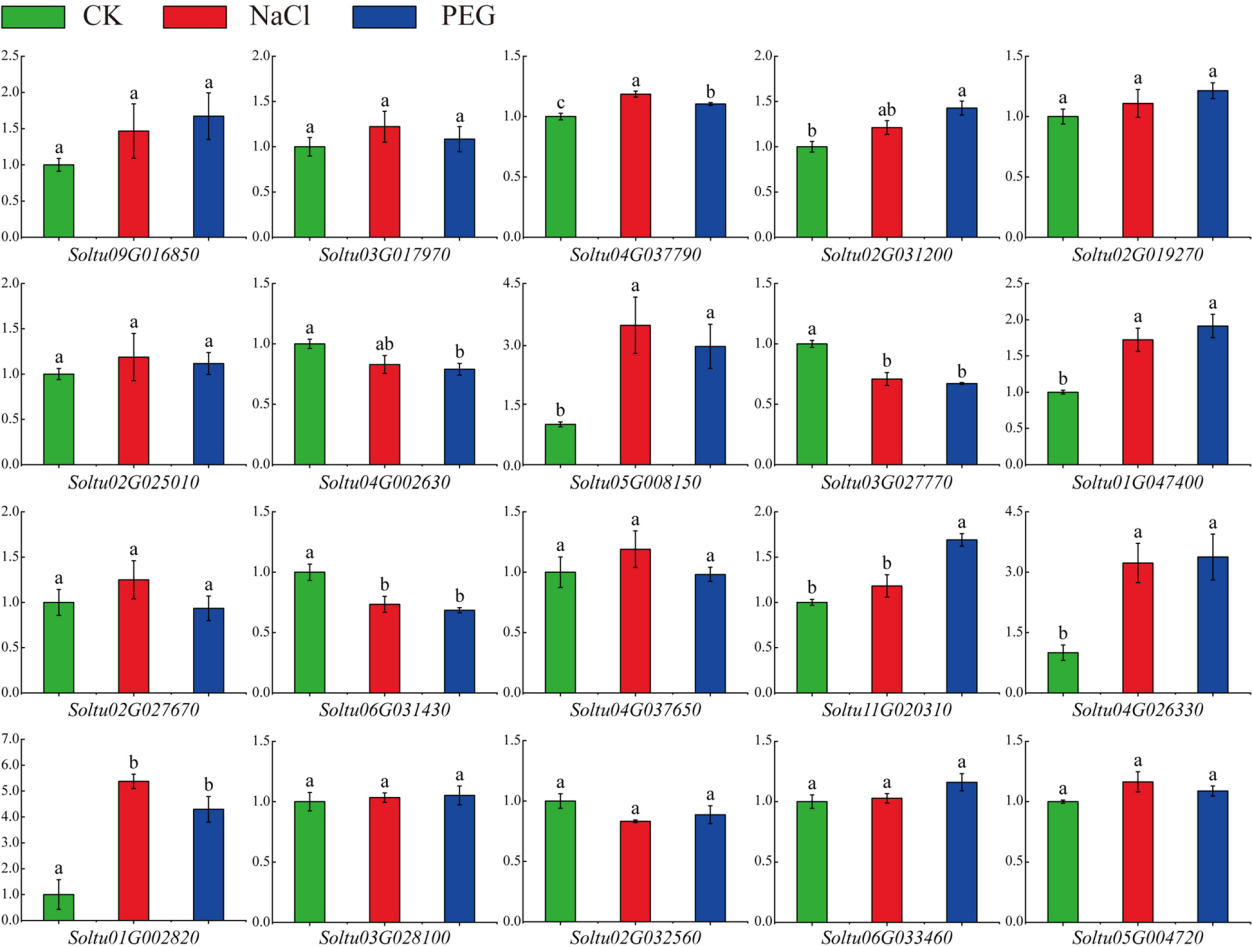
The 20 *StPUB* gene expression patterns in C98 under 24-h NaCl and PEG stresses. Results are representative of mean ± standard error of the mean (SEM) from 3 independent biological replicates. Standard errors (SEs) are displayed as bars on top of diverse columns. Diverse letters on top of the bars indicate significant differences at *P* < 0.05

Based on the results of RNA-seq in DM, we selected 20 differentially expressed StPUBs (|log_2_FC|> 1 and FPKM > 5) and analyzed the expression patterns of these genes in the tetraploid potato plantlets ‘CIP397098.12’ (CIP98) under NaCl (NaCl 100 mM, 24 h) and PEG (PEG-6000 10% w/v, 24 h) treatments (Fig. 8). After treatment with NaCl, *Soltu05G008150*, *Soltu01G002820*, and *Soltu04G026330* were significantly up-regulated, with the relative expression of *Soltu05G008150*, *Soltu01G002820*, and *Soltu04G026330* was 3.46, 5.37, and 3.23 times higher than that of the control. Four *StPUBs* (*Soltu05G008150*, *Soltu01G002820*, *Soltu01G047400*, and *Soltu04G026330*) responded significantly to PEG stress, which was 2.94, 4.30, 2.09, and 3.38 times higher than that of control, respectively (Fig. 8). The results indicated that these four *StPUB* genes might be related to abiotic stress response in tetraploid potato.

### *StPUB* gene expression patterns under drought stress in different potato cultivars

**Fig. 9:**
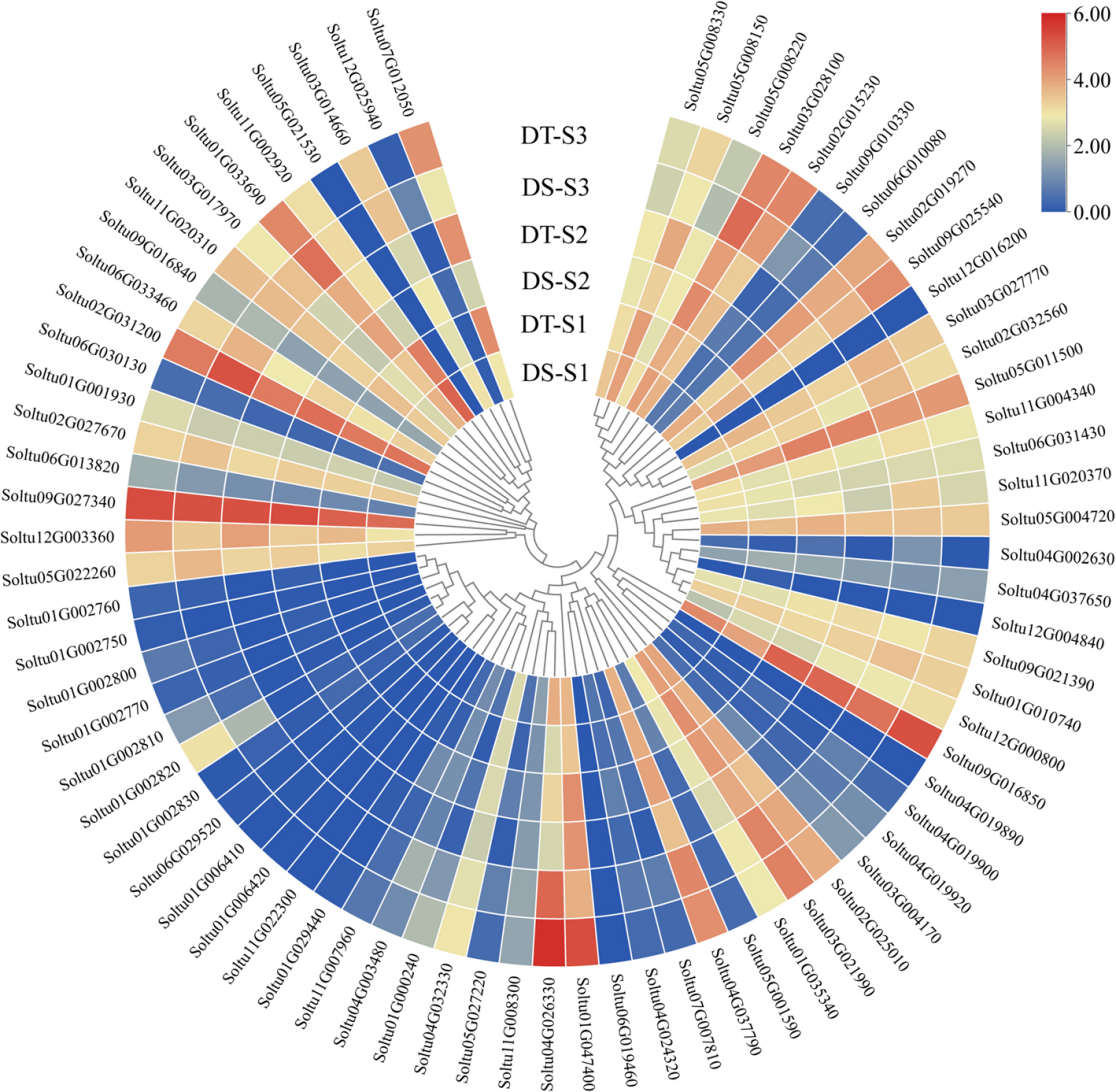
The expression profiles of *StPUB* genes under drought stress at S1, S2 and S3 stage in DS and DT. Log2 mean of FPKM value for all genes was used to plot the color scale

In order to further investigate whether these candidate *StPUB* genes were also in drought stress response in tetraploid cultivars, the DS cultivar and DT cultivar were subject to drought stress treatment. As revealed by RNA-seq analysis, three *StPUB* genes were not detected in the tetraploid cultivar DS and DT, and the expression of 20 *StPUB* genes was less than 1. Six *StPUBs* (*Soltu01G000240*, *Soltu01G002810*, *Soltu01G002820*, *Soltu01G047400*, *Soltu04G026330*, and *Soltu11G020370*) were up-regulated or down-regulated (FPKM > 1 and |log_2_FC|> 1) in DT under drought stress compared to DS. Among them, 4 *StPUBs* (*Soltu01G000240*, *Soltu01G002810*, *Soltu01G002820*, and *Soltu01G047400*) were not differentially expressed at S1 and S2 stage, whereas up-regulated in DT cultivar at S3 stage under drought stress. One *StPUB* gene (*Soltu11G020370*) was only down-regulated in S3. It is of note that the expression of one *StPUB* (*Soltu04G026330*) increased gradually with the aggravation of drought stress, which down-regulated at the flowering stage and high expressed at the falling flowering stage (log_2_FC = 0.90) (Figs. 8 and 9, Table S8).

Combined with mannitol and drought stresses analyses, three *StPUB* candidate genes (FPKM > 5 and |log_2_FC|> 1) were selected. Among them, two *StPUBs* (*Soltu01G002820* and *Soltu01G047400*) were up-regulated in DM potato under mannitol stress, whereas up-regulated in DT at S3 stage. One *StPUB* (*Soltu04G026330*) was up-regulated under mannitol stress in DM, and down-regulated in DT at S1 stage, and highly expressed in DT at S3 stage under drought stress. In addition, all these three *StPUB* genes only contained the U-box domain.

**Fig. 10:**
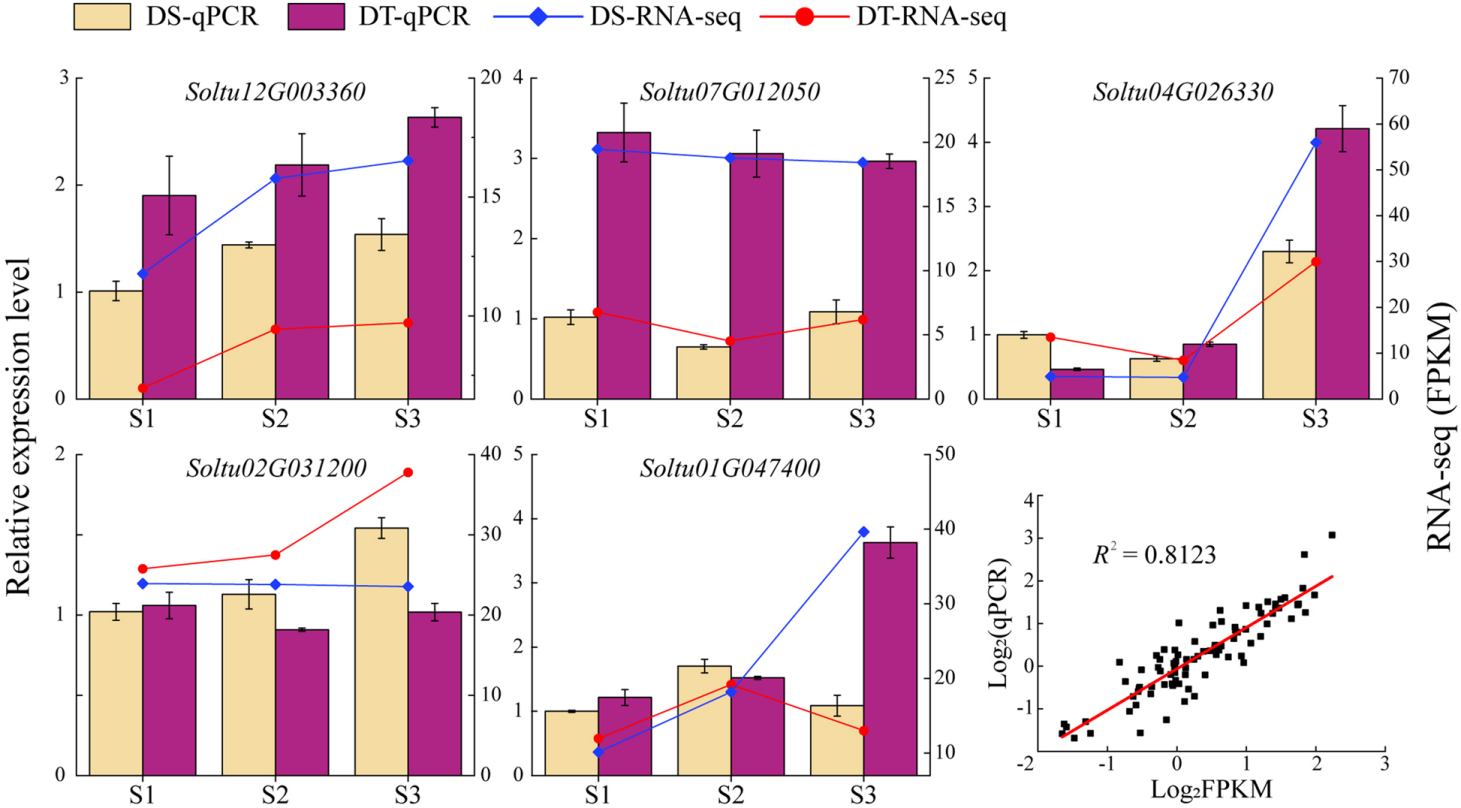
The qPCR expression analyses of five *StPUB* genes in DS cultivar and DT cultivar. S1, S2, and S3 represent the early flowering, full-blooming, and flower-falling stages, respectively.Data represent the mean of three biological replicates ± standard error of the mean (SEM)

The five *StPUB* genes with differentially expressed or relatively high expression levels in drought-tolerant cultivar (DT) were selected as targets to verify the reliability of the RNA-seq dataset by qPCR. The results showed that the qPCR expression patterns consistent with the RNA-Seq dataset (Fig. 10), the linear regression equations (*y* = 0.9687 *x*-0.0635) between the RNA-seq dataset and qPCR showed a high correlation (*R*^2^ = 0.8123).

### Phenotypic and physiological properties of *StPUB25* transgenic plants under drought stress

We cloned the cDNA sequence of *Soltu01G47400* from the DT cultivar (QS3). The cDNA sequence of *Soltu01G47400* was 98.6% similar to the reference genome sequence Version 6.1 (Fig. S2). Through phylogenetic analysis, it was found that *Soltu01G47400* and *AtPUB25* (*AT3G19380*) were orthologous genes, so *Soltu01G47400* was named *StPUB25*.

**Fig. 11:**
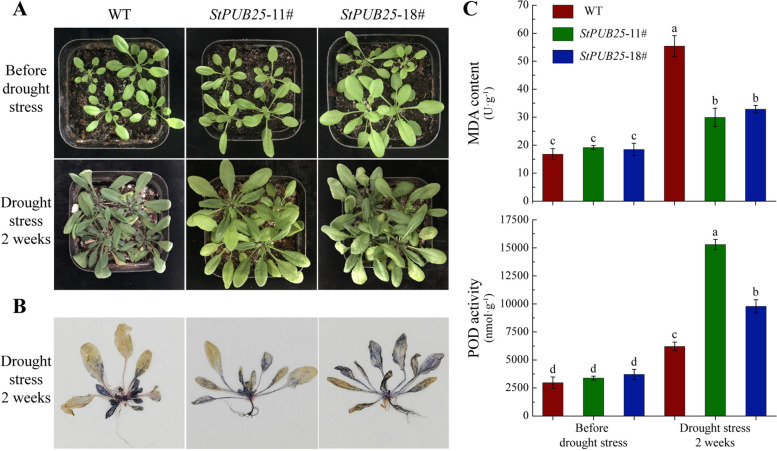
Functional identification of StPUB25 in transgenic *Arabidopsis*. Growth phenotypes of *StPUB25* transgenic plants and WT under drought stress **A** NBT staining of *StPUB25* transgenic plants and WT under drought stress **B** Determination of MDA content and POD activity in *StPUB25* transgenic plants and WT **C** Data represent the mean of three biological replicates ± standard error of the mean (SEM). Different letters represent significant difference at *p* < 0.05

In order to elucidate the function of StPUB25 in drought stress, we generated transgenic plants harboring *StPUB25* driven by the CaMV35S promoter (Fig. S3). Then, four-week-old plants were treated with drought stress (withholding water for two weeks). We observed the growth phenotypes of *StPUB25* transgenic plants and wild-type (WT) plants under normal water conditions and found no significant difference between them. After drought stress, the WT plants were significantly more damaged than the *StPUB25* transgenic lines. The leaves of the WT plants were dark green, and some leaves were withered and wilted. However, the leaves of the two *StPUB25* transgenic lines were still light green, and the growth phenotype was significantly better than that of the WT plants (Fig. 11-A).

To further clarify the effect of *StPUB25* on the physiological indices of transgenic plants under drought stress, we performed NBT (nitroblue tetrazolium) staining and measured MDA content and POD activity in WT and *StPUB25* transgenic plants under drought stress. The results of NTB staining showed that StPUB25 transgenic plants had significantly lower ROS (reactive oxygen species) content than WT plants, especially in young leaves (Fig. 11-B). After drought stress, the MDA content in control plants was significantly (*p* < 0.05) higher than that in *StPUB25* transgenic lines, 1.85 and 1.68 times higher than in line 11# and line 18#, respectively (Fig. 11-C). Furthermore, the POD activity was also significantly increased in all plants after drought stress, and the POD activity was significantly higher in both transgenic lines than in WT, especially line 11# (Fig. 11-C). Thus, we suggest that *StPUB25* transgenic lines are more tolerant to drought than WT plants, which may be due to their higher POD activity, thereby reducing plant damage by ROS.

## Discussion

Previous reports have shown that several PUBs have essential roles in plant growth, development, and stress responses [15, 20, 21, 25]. This study discovered altogether 74 genes encoding PUB proteins in potato, and their chromosomal location, conserved motifs, gene structures, and gene duplication events were analyzed. Besides, this study analyzed *StPUB* gene expression profiles within diverse tissues, along with the corresponding abiotic stress and hormone treatment responses in DM potato. Furthermore, *StPUB* gene expression patterns in DS and DT potato cultivars under drought stress were performed. This study provided valuable information to further understand the functions of the U-box genes in potato.

### Expansions of the *StPUB* gene family in potato

The distribution of U-box protein was uneven among different species [13]. So far, the U-box gene family is systematically discovered within multiple plants, with 91 in banana [15], 77 in rice [13], 62 in tomato [20], 61 in *Arabidopsis* [45], 56 PUBs in pomegranate [22], 99 PUBs in cabbage [23], 62 PUBs in Chinese white pear [24], 79 PUBs in corn, and 125 PUBs in Soybean (*Glycine max*) [25], and 56 in Grapevine [21] separately. Following our results, there were 74 *PUB* genes detected in potato, with a close number compared with other plants. The PUBs only account for a low percentage in plant E3s, but their numbers significantly increased than in yeast (2 members) and humans (21 members) [12, 14, 46], suggesting the PUBs may have diverged more functions in plants.

Gene duplication is the main factor for gene family expansion and evolution, possibly the critical factor driving gene functional diversification [47, 48]. According to our results, 12 duplication *StPUB* gene pairs were identified in potato, of which eight *StPUB* genes (8/74, 10.81%) were identified as tandem duplicated, and 14 *StPUB* genes (14/74, 18.92%) were identified as segmental duplicated. Interestingly, the duplication events mainly occurred in the C3, C5, C6, C7, and C8 subclasses, and there were five pairs of duplicated genes in the C6 subclass and four pairs of duplicated genes in the C8 subclass. These results suggested that the duplication events in the C6 and C8 subclasses were main major cause of expansion of the StPUB gene family.

### Analysis of StPUBs phylogenetic and evolution

An unroot phylogenetic tree was constructed to analyze relations between U-box proteins in potato, *Arabidopsis*, tomato, cabbage, rice, and corn. As a result, U-box was classified as eight subclasses (C1-C8). The U-box domain was found to be critical for the ubiquitination activity [49]. Hatakeyama et al. found that U-box deletion or conserved amino acid mutations inside abolished the ubiquitination effect [46]. Furthermore, some StPUB members contained the U-box domain and other domains, including ARM, WD40, and TPR (tetratricopeptide).

The ARM repeat is an approximately 40 amino acid tandem repeat, and it was originally discovered in *Drosophila melanogaster* protein β-catenin [50]*.* It has been reported that ARM repeats mainly mediate the interaction with substrates to be ubiquitinated [51]. Our study found that 25 (33.78%) StPUBs contained ARM domains, and 49 (66.22%) StPUBs had no ARM repeats, indicating that the StPUBs without ARM repeats may have different interactions with corresponding substrates relative to StPUBs that contain ARM repeats.

WD40 domain was originally found in the bovine *β*-transduction protein, containing a highly conserved glycine-histidine (GH) and tryptophan-aspartate (WD) 43 residue substrate for repeat orders [52]. WD40 has more interactions with other domains, acting as a scaffold for protein interactions or providing a platform to recruit different molecules to form functional complexes [53]. In this study, four StPUBs (Soltu04G024320, Soltu07G007810, Soltu09G027340, and Soltu12G003360) were identified, which contained U-box and WD40 domain. These StPUBs were not differentially expressed under abiotic stress, but two of them (Soltu09G027340 and Soltu12G003360) were highly expressed in multiple potato tissues. Whether they are involved in the development of specific tissues deserves further investigation.

Tetrapeptide repeat (TPR) motifs are protein–protein interaction modules that are present many functionally diverse proteins and contribute to specific interactions with chaperone proteins [54, 55]. Previous studies found that AtCHIP (AT3G07370) interacts with molecular chaperones such as Hsc70 through its TPRs and may therefore regulate the switch from molecular chaperon-assisted protein folding to proteasomal degradation. In this study, only one StPUBs (Soltu06G033460) contained three TPR motifs, which was homologous to AtCHIP. Soltu06G033460 may have similar functions as AtCHIP.

### Expression profile analysis of *StPUB* genes

Gene expression in specific tissues may be often adopted for preliminarily predicting associated activities [56]. In our study, 10 *StPUBs* were differentially expressed in potato stolons. Among them, the expression level of five StPUBs was also higher in tubers. Previous studies found that the PUBs have interesting functions in plant development. For example, *pub4* mutants showed higher levels of cell proliferation in both the root tip meristem and shoot tip meristem [57]. Srijani et al. [27] have found that the interaction between ARK2 and AtPUB9 leads to AtPUB9 accumulation in autophagosomes, ARK2-PUB9 signaling module is involved in regulation of lateral root development via selective autophagy. In addition, several PUBs are also involved in the regulation of plant flowering [28, 58]. However, whether PUBs are involved in potato tuber development has not been reported. Whether the above StPUBs specifically expressed in stolons and tubers are involved in potato tuber development needs further investigation.

U-box protein has been previously suggested to participate in different abiotic stress responses [25, 40, 59]. In this work, 11, 24, and 12 *StPUBs* were differentially expressed (FPKM > 1, |log_2_FC|> 1) under salt stress, drought stress, and heat stress, separately. Consistent conclusions have also been found in crops such as grapevine, banana, and rice [13, 15, 21], suggesting the critical effects of U-box gene family on plant responses to various abiotic stresses. Generally speaking, one cluster members possibly has the same evolutionary origins and conserved functions, so the known member functions in the same cluster can be used to infer the functions of other members [60]. In previous studies, *AtPUB18* and *AtPUB19* are reported to negatively regulate ABA-dependent stomatal closure and responses to drought stress [32]. *Soltu05G008150* showed homology to *AtPUB18*, and its expression increased upon drought and salt stresses within DM, indicating the close functions of *Soltu05G008150* to *AtPUB18* under abiotic stress. Zhao et al. [61] found that the *AtPUB22* and *AtPUB23* adversely modulated drought resistance partially through promoting PYL9 degradation of ABA receptors in *Arabidopsis*. *Soltu01G002820* was clustered with AtPUB22 and AtPUB23 in one cluster. It was up-regulated under salt and drought stress and down-regulated under ABA treatment. Notably, *Soltu01G002820* was also up-regulated in DT at the S3 stage. These results suggested that *Soltu01G002820* may have a similar function to *AtPUB22* and *AtPUB23*, which may be involved in regulating potato tolerance to drought stress through an ABA-dependent pathway. Adler et al*.* [40, 62] found that *AtPUB46* and *AtPUB48*’s single homozygous mutants exhibited hypersensitivity to drought stress, and overexpress of *AtPUB46* enhanced tolerance to drought and oxidative stress in transgenic *Arabidopsis*. *Soltu09G016850* was clustered with *AtPUB46* and *AtPUB48*, up-regulated under salt and drought stress in DM, indicating *Soltu09G016850* may also be related to abiotic stress responses in potato. Tang et al. [63] found that *StPUB27* may negatively regulate the tolerance of transgenic potato to drought by mediating stomatal conductance. According to the blastn analysis, the *StPUB27* found by Tang et al. was *Soltu03G028100*, which only differentially expressed in DM, but not differentially expressed in tetraploid potato under abiotic stress. This result may be related to the different ways of handling. Furthermore, we found that three *StPUB* genes, which have not been reported in potato, were significantly up-regulated not only under NaCl and PEG stresses in the tetraploid potato plantlets (C98) but also in DT cultivar under drought stress. These three PUB genes may be involved in the regulation of potato tolerance to drought stress. Their functions deserve further investigation.

### Functions of *StPUB25* in plant

Previous studies have shown that PUB25 involves plant growth, development, and responses to biotic/abiotic stresses. Li et al. [64] found that AtPUB25 is the main target mediating the action of petal-specific growth-promoting transcription factor RABBIT EARS (RBE) during petal development, which inhibits petal growth by limiting the cell proliferation phase. Wang et al. [65] found that the *AtPUB25* lead to MYB15 degradation and improve the expression of CBF under cold stress, thereby enhancing the freezing tolerance in *Arabidopsis*. Moreover, PUB25 maintains the protein homeostasis of BIK1 by interacting with the E3 ubiquitin ligase RGLG1/2 to regulate immune signaling in plants [66]. This study found that overexpression of *StPUB25* significantly increased POD activity, reduced ROS and MDA accumulation, and enhanced drought stress tolerance in transgenic *Arabidopsis*. However, the underlying mechanisms need to be further investigated.

## Conclusion

The present work detected altogether 74 StPUBs in potato genome, which showed uneven distributions on ten chromosomes. Based on gene structures and motifs with high conservation degrees, this work phylogenetically classified StPUBs as eight different subclasses. Segmental and tandem duplication events have essential effects on the *StPUB* gene family expansion. As revealed by synteny analysis, there were 40, 58, 28, 7 and 8 orthologous *StPUB* genes to *Arabidopsis*, tomato, cabbage, rice, and corn, separately. Besides, such gene pairs evolved under purifying selection. Some *StPUB* genes were expressed in a tissue-specific manner, and nine *StPUBs* exhibited specific expression in stolons, of which two *StPUBs* exhibited specific expression in tuber, which may be involved in stolon formation and tuber development. In addition, *StPUB* genes displayed diverse expression profiles in DM and tetraploid potato (DS and DT) under salt, mannitol, heat, and drought stresses. It is noteworthy that three *StPUB* genes involved in response to abiotic stress, which may enhance the drought stress resistance of potato. Furthermore, this study found that overexpression of *StPUB25* significantly increased POD activity, reduced ROS and MDA accumulation, and enhanced drought stress tolerance in transgenic *Arabidopsis*. Our findings should provide valuable information for the characteristics and biological function of the StPUB gene family in potato.

## Materials and methods

### Genome identification of StPUBs in potato

For identifying *StPUB* genes in potato, this work obtained the potato nucleotide and amino acid (aa) sequences in Potato Genome Sequencing Consortium database (PGSC, http://spuddb.uga.edu/dm_v6_1_download.shtml, version 6.1). To identify the *StPUB* genes in potato, two methods were used separately and then combined: (1) HMM profiles for the U-box domain (PF04564) in were obtained in the Pfam database (http://pfam.xfam.org/family/PF04564). HMMER3.1 software (http://hmmer.org/down load.html) was later employed to identify StPUB protein. (2) This study also acquired sequences of those 61 known *Arabidopsis* U-box (AtPUB) proteins in the *Arabidopsis* information resource (https://www.arabidopsis.org/browse/genefamily/pub.jsp). StPUBs were searched using the BLASTP [67] according to 61 AtPUB aa sequences (https://www.arabidopsis.org/browse/genefamily/pub.jsp) with an Evalue ≤ 1e^−5^. The redundant protein sequences were removed, then NCBI Conserved Domain Data (CDD) and SMART (http://smart.embl-heidelberg.de/) were utilized to verify all the candidate StPUBs, and members without the U-box domain were deleted.

### Analysis of sequences and structures

ExPasy site (http://web.expasy.org/protparam/) [68] was employed for predicting aa number, theoretical isoelectric point (pl), and molecular weight (MW). Besides, the MEME program (http://meme.nbcr.net/meme/tools/meme) was utilized to detect the conserved motif in all StPUBs [69]. Parameters were set with optimum width of 6 to 50 aa, one motif involving repetitions at all numbers, with at most 20 motifs altogether. The full-length coding sequence (CDS) was compared against relevant genomic DNA (gDNA) sequences to construct gene structure of *StPUBs.* Gene Structure Display Server (GSDS 2.0, http://gsds.cbi.pku.edu.cn/) was used to obtain gene structures of the *StPUBs* [70]. The subcellular localization of StPUBs were predicted using CELLO V. 2.5 (http://cello.life.nctu.edu.tw/) [71].

### Chromosomal localization and gene duplication

Information about the chromosomal location of each *StPUB* gene was retrieved from the PGSC. This study plotted the map showing *StPUB* gene distribution using Mapchart software (version 2.32, http://www.wageningenur.nl/en/show/Mapchart.htm) [72]. In addition, duplicates in *StPUB* genes in potato were analyzed by MCScanX [73], then PUB gene synteny among potato, tomato, *Arabidopsis*, cabbage, rice, and corn were identified. The figures were drawn with the use of Circos v0.69 [74]. For better analyzing gene duplicates, KaKs Calculator 2.0 [75] was applied to determine Ks (synonymous) and Ka (non-synonymous) for every duplicated PUB gene pair.

### Phylogenetic analysis and classification of PUBs

This work adopted full-length amino acid sequences of the 74 StPUBs, 61 AtPUBs, 62 SlPUBs, 77 OsPUBs, 79 ZmPUBs, and 99 BoPUBs to conduct phylogenetic analysis. The ClustalW algorithm was used for multiple sequence alignments under default parameters. Then this study imported those aligned sequence data into MEGA7.0 program [37] and built an unrooted maximum likelihood phylogenetic tree under 1000 bootstraps.

### Plant materials and treatments

In drought stress assay, the DS cultivar ‘Atlantic’ (A) and the DT cultivar ‘Qingshu No.9’ (Q) were planted in the field covered by one rainproof shelter in Dingxi Academy of Agricultural Science in Dingxi, Gansu Province, China. This assay was of the randomized block design, three replicates were set under every treatment, and ten plants were planted in every experiment unit. For the first 4 weeks, each experimental unit was irrigated with 30 mm water. After seedling, the plants were not watered for the rest growth period. Leaves from three plants of every experiment were collected and combined with being one sample at early flowering (S1), full-blooming (S2), as well as flower-falling (S3) stages, respectively. Samples were frozen within liquid nitrogen and preserved at − 80 °C until subsequent analysis.

Regarding the NaCl-/PEG-treated experiment, the tetraploid potato plantlets ‘CIP397098.12’ (CIP98) from the International Potato Center (CIP) was used as material for qPCR. We propagated CIP98 Plantlets onto the Murashige and Skoog (MS) liquid medium that contained 3% sucrose (w/v, pH 5.8 ± 1) in-vitro, then cultivated them within the incubator under 23 ± 2 °C, 16-h/18-h photoperiod and 200 μmol m^−2^ s^−1^ irradiation for 20 days. Then, 100 mM NaCl and 10.0% PEG-6000 (w/v) were added to treat plantlets for a 24-h period. We put five plantlets within every triangular flask. Later, the whole plant from all these three triangular flasks plants for each replicate was collected. Each sample was subject to liquid nitrogen freezing at once, followed by preservation in − 80 °C to extract total RNA and analyze gene levels.

### RNA extraction and quantitative real-time PCR

This work utilized RNA extraction kit (Tiangen DP419, Beijing, China) for isolating total RNA. Later, agarose gel electrophoresis (AGE) was conducted to detect RNA integrity, while the Nanodrop ND-2000 spectrophotometer (Nanodrop Technologies, USA) was employed to detect RNA content. Besides, the FastKing RT kit that contained gDNase (Tiangen KR116, Beijing, China) was adopted for cDNA synthesis. SuperReal PreMix Plus kit (SYBRGreen FP205, Tiangen, Beijing, China) was applied in qPCR on Bio-Rad CFX96 (Bio-Rad, Hercules, CA, USA). Conditions of the PCR procedure were shown below, 30-s under 95 °C; 5-s under 95 °C and 30-s under 60 °C for 40 cycles; and melting curve detection under 65–95 °C. The assay was carried out in triplicate independently. 2^−ΔΔCt^ approach was adopted for determining the relative expression levels of genes, with *StEF-1α* (AB061263) being an internal control. Table S9 displays the primers.

### Expression patterns of *StPUBs* in potato

According to Illumina RNA-seq data obtained in PGSC (The Potato Genome Sequencing et al., 2011), this study examined *StPUB* gene expression patterns in 14 DM potato tissues (stamens, leaves, stolons, shoots, tubers, roots, callus, carpels, petioles, petals, flowers, sepals, immature fruit, mature fruit), and within *in-vitro* whole plant exposed to abiotic stresses (heat treatment at 35 °C; salt treatment with 150 mM NaCl; mannitol-mediated drought stress with 260 μM Mannitol) and hormone treatments (abscisic acid-ABA: 50uM, 24 h; benzylaminopurine-BAP: 10uM, 24 h; gibberellic acid-GA3: 50uM, 24 h; indole acetic acid-IAA: 10uM, 24 h). The heatmap was plotted with the TBTools program [76].

Moreover, based on the RNA-seq analysis, *StPUB* gene expression levels in 3 stages of tetraploid potato of DS cultivar and DT cultivar under drought stress were examined. Biomarker Technologies Corporation (Beijing, China) was responsible for Illumina sequencing. Raw reads were trimmed to eliminate pollutants, adapters, uncertain bases N and phred scores < 20, to obtain high quality clean reads. Through adopting Bowtie v2.2.9 software, this work aligned cleaned data against the PGSC_DM_v6.1 gene models obtained in Solanaceae Genomics Resource at Michigan State University (http://solanaceae.plantbiology.msu.edu/pgsc_download.shtml). Later, we determined the mapped clean read count (by fragment) and performed differential analysis on these clean reads by adopting the edge R package (http://www.r-project.org/). Differentially expressed genes (DEGs) were later screened upon the thresholds of false discovery rate (FDR) < 0.05 and |log_2_ fold change (FC) |> 1. At last, we submitted raw data to NCBI (Project ID PRJNA541096).

### Plasmid construction and plant transformation

The coding sequence of *Soltu01G47400* was amplified using cDNA from leaves of the DT cultivar (Q) at S3 stage and the following designed primers: *Sotul01G47400*-F: 5′ATGCCTGGAAGTTTAGACCCTTTGG-3′, *Sotul01G47400*-R: 5′-TCAAAACGGAACGACGTCGC-3'. Gene amplification was achieved by PCR using PrimeSTAR HS DNA Polymerase (TaKaRa, Japan), and the cDNA of *Sotul01G47400* was cloned into the plant expression vector pNC-Cam2304-MCS35S by nimble clonin. The constructs were verified by sequencing and transformed into *Agrobacterium* strain GV3101. The pNC-Cam2304MCS35S-*Soltu01G47400* construct was introduced into *Arabidopsis thaliana* ecotype Columbia (WT) using the *Agrobacterium*-mediated floral dip method [77].

### Drought stress of transgenic *Arabidopsis*

*StPUB25* transgenic *Arabidopsis* and WT were cultured in a climate chamber under the following conditions: 12 h light /12 h dark photoperiod, 22 ± 2 ℃, and 65% relative humidity. After 4 weeks of growth, plants with consistent growth were selected for drought treatment. For drought-stress treatment, all plants were grown for two weeks without water irrigation. The NBT staining was performed using NBT Kit (G1023-100ML, Servicebio Technology CO, Wuhan, China). The POD activity and MDA content were measured using Micro Peroxidase Assay Kit and Malondialdehyde Content Assay Kit (Cat# BC0095 and Cat# BC0025, Solarbio Biochemical Assay Division, Beijing, China). All experiments were performed with three biological replicates.

### Statistical analysis

Data significance analysis was performed using IBM SPSS Statistics 26 software. Bars with different letters are significantly different at *P* < 0.05, according to One-way ANOVA and least significant difference (LSD). Data are shown as means (± SE) from three separate assays.

### Supplementary Information

**Additional file 1:** **Table S1.** The 74 StPUBs characteristics.

** Additional file 2:** **Table S2.** The conserved motifs identified by MEME among StPUBs.

** Additional file 3:** **Table S3.** The conserved motifs identified by MEME among PUBs in the multi-species.

** Additional file 4:** **Table S4.** Ka/Ks values of StPUB genes with tandem and segmental duplications.

** Additional file 5:** **Table S5.** Ka/Ks values of syntenic relations between potato and tomato.

** Additional file 6:** **Table S6.** Ka/Ks ratios of the syntenic relationships between potato and Arabidopsis.

** Additional file 7:** **Table S7.** Ka/Ks ratios of the syntenic relationships between potato and cabbage.

** Additional file 8:** **Table S8.** Ka/Ks ratios of the syntenic relationships between potato and rice.

** Additional file 9:** **Table S9.** Ka/Ks ratios of the syntenic relationships between potato and corn.

** Additional file 10:** **Table S10.** The RNA-seq data for StPUB genes in diverse tissues.

** Additional file 11:** **Table S11.** The RNA-seq data for StPUB genes under abiotic stresses and hormone treatments.

** Additional file 12:** **Table S12.** The FPKM value of StPUB genes under drought stress in different potato cultivars (DT and DS).

** Additional file 13:** **Table S13.** Primers utilized in qPCR.

** Additional file 14:** **Figure S1.** Comparative analysis of conserved motifs identified by MEME. (A) The conserved motifs in potato pubs. (B) The conserved motifs in PUBs among potato and other plants.

** Additional file 15:** **Figure S2.** The sequence alignment of sequencing results and reference genes. The similarity  between the sequencing results and the reference genome was 98.6%.

** Additional file 16:** **Figure S3.** Identification of transgenic plants. Marker: 1500 bp DNA marker, +: pNC-Cam2304MCS35S-StPUB25 plasmid, -: DD H2O, WT: wild-type Arabidopsis, 11# and 18# were overexpress StPUB25 transgenic Arabidopsis.

## Data Availability

The gene expression data in the doubled-monoploid potato (DM) were downloaded from the PGSC website with accession number SRA030516 in NCBI. The RNA-seq on the ‘Atlantic’ and the ‘Qingshu NO.9’ at three stages were performed by Biomarker Technologies Corporation (Beijing, China), and the raw data was submitted to NCBI (Project ID PRJNA541096).
