# Supplementary material for: Genome-wide analysis of the U-box E3 ligases gene family in potato (Solanum tuberosum L.) and overexpress StPUB25 enhance drought tolerance in transgenic Arabidopsis
Source: BMC Genomics. 2024 Jan 2;25:10. doi: 10.1186/s12864-023-09890-5 (PMC10759479; doi:10.1186/s12864-023-09890-5)
Supplement: Supplementary file 14 — Additional file 14: Figure S1. Comparative analysis of conserved motifs identified by MEME. (A) The conserved motifs in potato pubs. (B) The conserved motifs in PUBs among potato and other plants. [file 12864_2023_9890_MOESM14_ESM.pdf]

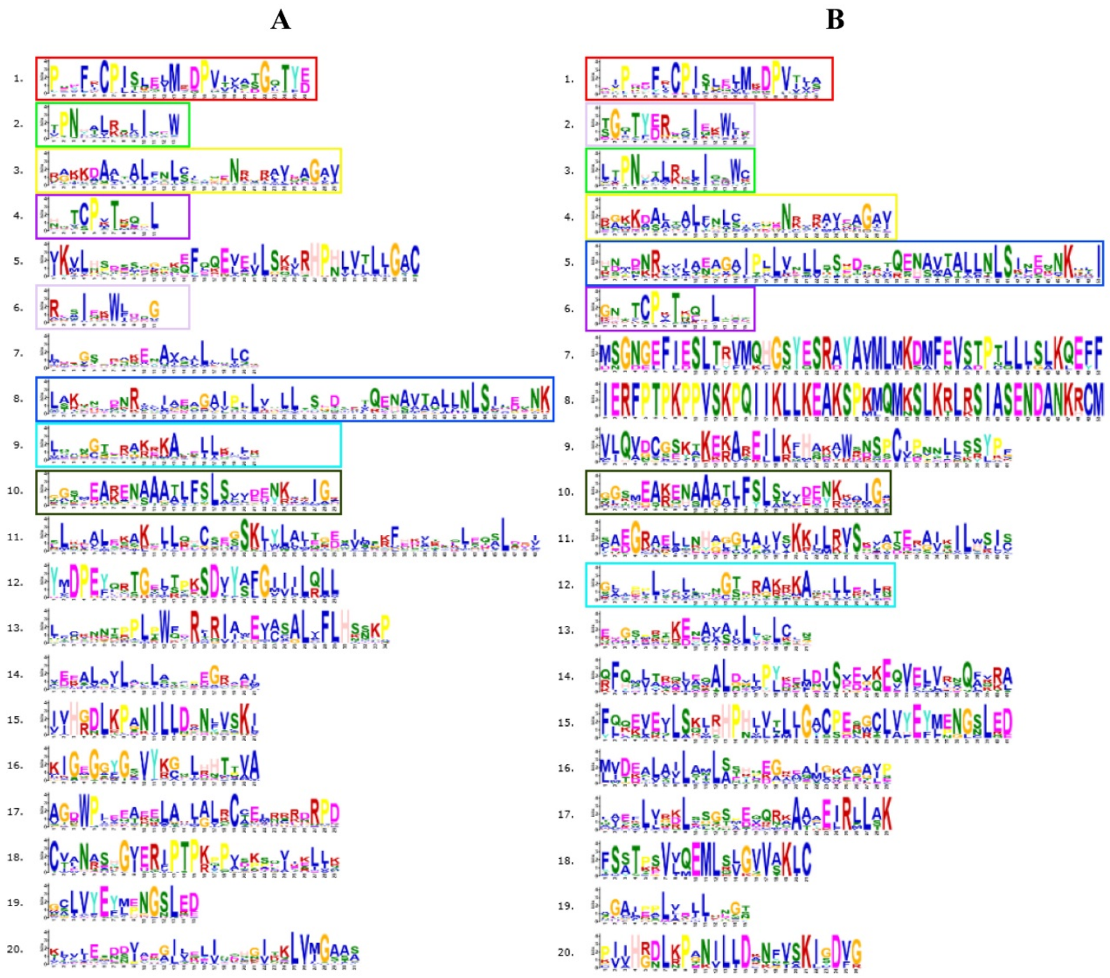

Figure S1. Comparative analysis of conserved motifs identified by MEME. (A) The conserved motifs in potato pubs. (B) The conserved motifs in PUBs among potato and other plants.
