# Supplementary material for: Genome-wide analysis of the U-box E3 ligases gene family in potato (Solanum tuberosum L.) and overexpress StPUB25 enhance drought tolerance in transgenic Arabidopsis
Source: BMC Genomics. 2024 Jan 2;25:10. doi: 10.1186/s12864-023-09890-5 (PMC10759479; doi:10.1186/s12864-023-09890-5)
Supplement: Supplementary file 15 — Additional file 15: Figure S2. The sequence alignment of sequencing results and reference genes. The similarity between the sequencing results and the reference genome was 98.6%. [file 12864_2023_9890_MOESM15_ESM.pdf]

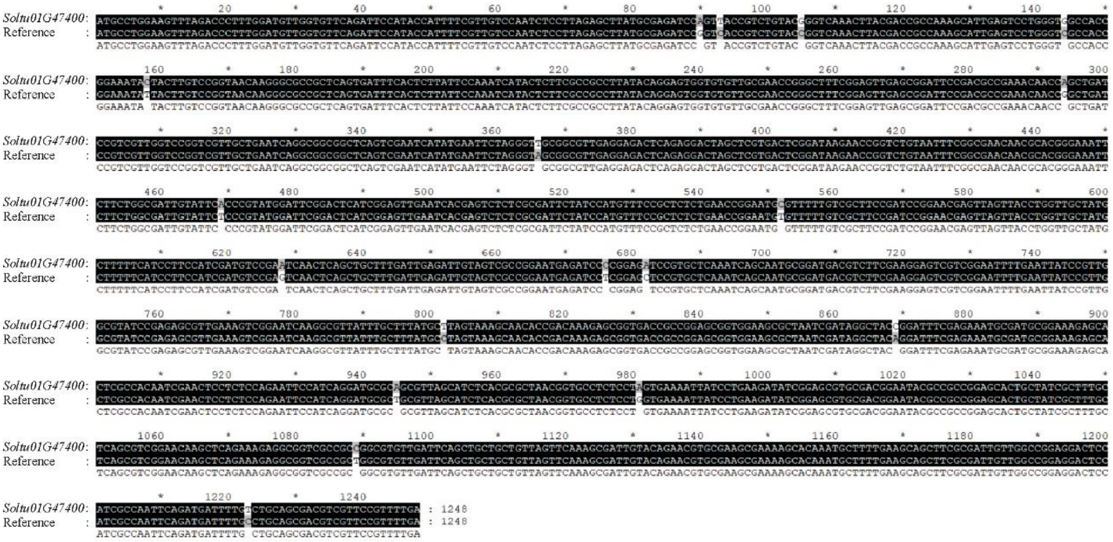

Figure S2. The sequence alignment of sequencing results and reference genes. The similarity between the sequencing results and the reference genome was 98.6%.
