# Supplementary material for: Genome-wide analysis of the U-box E3 ligases gene family in potato (Solanum tuberosum L.) and overexpress StPUB25 enhance drought tolerance in transgenic Arabidopsis
Source: BMC Genomics. 2024 Jan 2;25:10. doi: 10.1186/s12864-023-09890-5 (PMC10759479; doi:10.1186/s12864-023-09890-5)
Supplement: Supplementary file 16 — Additional file 16: Figure S3. Identification of transgenic plants. Marker: 1500 bp DNA marker, +: pNC-Cam2304MCS35S-StPUB25 plasmid, -: DD H2O, WT: wild-type Arabidopsis, 11# and 18# were overexpress StPUB25 transgenic Arabidopsis. [file 12864_2023_9890_MOESM16_ESM.pdf]

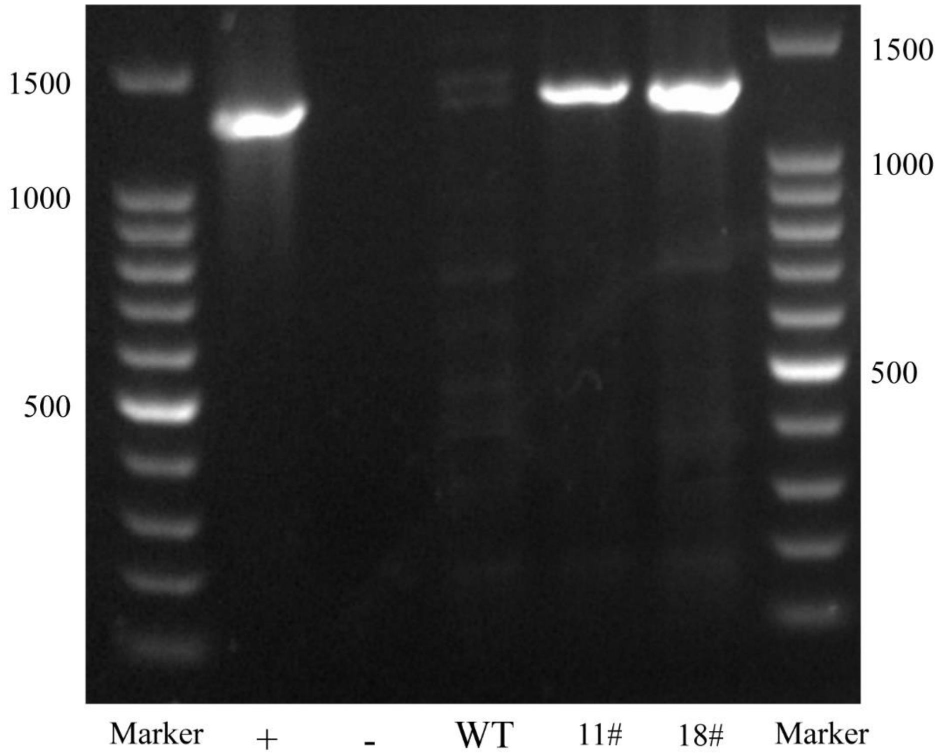

Figure S3. Identification of transgenic plants. Marker: 1500 bp DNA marker, +: pNC-Cam2304MCS35S-*StPUB25* plasmid, -: DD H_2O, WT: wild-type *Arabidopsis*, 11# and 18# were overexpress *StPUB25* transgenic *Arabidopsis*.
